# Impact of Hydrostatic Pressure Variations Caused by Height Differences in Supine and Prone Positions on Fractional Flow Reserve Values in the Coronary Circulation

**DOI:** 10.1155/2019/4532862

**Published:** 2019-09-02

**Authors:** Yoshitaka Kawaguchi, Kazuki Ito, Humihiko Kin, Yusuke Shirai, Ayako Okazaki, Keisuke Miyajima, Tomoyuki Watanabe, Mariko Tatsuguchi, Yasushi Wakabayashi, Yuichiro Maekawa

**Affiliations:** ^1^Department of Cardiology, Seirei Mikatahara General Hospital, Hamamatsu, Shizuoka, Japan; ^2^Internal Medicine III, Hamamatsu University School of Medicine, Hamamatsu, Shizuoka, Japan

## Abstract

**Objectives:**

To examine the influence of hydrostatic pressure on fractional flow reserve (FFR) in vivo.

**Background:**

Systematic differences in FFR values have been observed previously in the left anterior descending artery (LAD), left circumflex artery (LCX), and right coronary artery (RCA). It has been suggested that as the hydrostatic pressure variations caused by the height differences between the catheter tip (mean aortic pressure (Pa)) and pressure-wire sensor (mean distal intracoronary pressure (Pd)) are small, intracoronary pressure need not be corrected.

**Methods:**

Resting Pd/Pa and FFR values in 23 patients (27 lesions) were measured and compared in supine and prone positions. These values were corrected by hydrostatic pressure influenced by height levels and compared. Height differences between Pa and Pd were calculated using coronary computed tomography angiographies.

**Results:**

In LAD, resting Pd/Pa and FFR values were significantly higher in the prone position than in the supine position (0.97 ± 0.05 vs 0.89 ± 0.04, *P* < 0.001 (resting Pd/Pa); 0.81 ± 0.09 vs 0.72 ± 0.07, *P* < 0.001 (FFR)). Conversely, in LCX and RCA, these values were significantly lower in the prone position (LCX: 0.93 ± 0.03 vs 0.98 ± 0.03, *P* < 0.001 (resting Pd/Pa); 0.84 ± 0.05 vs 0.89 ± 0.04, *P* < 0.001 (FFR); RCA: 0.91 ± 0.04 vs 0.98 ± 0.03, *P*=0.005 (resting Pd/Pa); 0.78 ± 0.07 vs 0.84 ± 0.07, *P*=0.019 (FFR)). FFR values corrected by hydrostatic pressure showed good correlations in the supine and prone positions (*R*^2^ = 0.948 in LAD; *R*^2^ = 0.942 in LCX; *R*^2^ = 0.928 in RCA).

**Conclusions:**

Hydrostatic pressure variations due to height levels influence intracoronary pressure measurements and largely affect resting Pd/Pa and FFR, which might have caused systematic differences in FFR values between the anterior and posterior coronary territories.

## 1. Introduction

Functional assessment of coronary artery stenoses using coronary artery pressure wires is an important diagnostic tool in the catheterization laboratory. Fractional flow reserve (FFR) is calculated as the ratio of distal intracoronary pressure to the proximal intracoronary pressure during pharmacologically induced hyperemia [1]. When compared with angiography-guided percutaneous coronary intervention (PCI), FFR-guided PCI has been shown to significantly improve patient outcomes and be cost-effective and is currently considered the gold standard for identifying the hemodynamic severity of coronary artery stenosis [2–8]. Recently, systematic differences in FFR values between the anterior and posterior coronary territories have been observed: the left anterior descending artery (LAD) supplying the anterior coronary territories had lower FFR values, whereas the left circumflex artery (LCX) and right coronary artery (RCA) with posterior coronary territories had higher FFR values in the supine position [9]. A reverse mismatch in LAD and mismatch in LCX and RCA have been reported [10–12]. The influence of hydrostatic pressure on the intracoronary pressure measurements was recently demonstrated in vitro [13]. Hydrostatic pressure variations due to height levels between the catheter tip and distal pressure sensor influence intracoronary pressure measurements and affect FFR values [13]. The hydrostatic pressure might explain these findings. In this study, we sought to analyze the influence of hydrostatic pressure on intracoronary pressure measurements in vivo by artificial modification of the height difference between the distal pressure sensor and catheter tip.

## 2. Methods

### 2.1. Ethics Statement

This study protocol was approved by the ethics committee at our institution and conducted in accordance with the guidelines of the Declaration of Helsinki. Written informed consent was obtained from all patients.

### 2.2. Study Population

Patients who were suspected of having ischemic heart disease and underwent cardiac computed tomography (CT) with confirmed stenosis were prospectively enrolled from September 2017 to November 2018. Patients with acute coronary syndromes, those who did not undergo cardiac CT, and those whose puncture sites were not radial arteries were excluded. Patients with pressure wires that were difficult to manipulate due to tortuous vessels in the supine position did not undergo another measurement in the prone position, as judged by the operator.

### 2.3. Intracoronary Pressure Measurements

**Figure 1:**
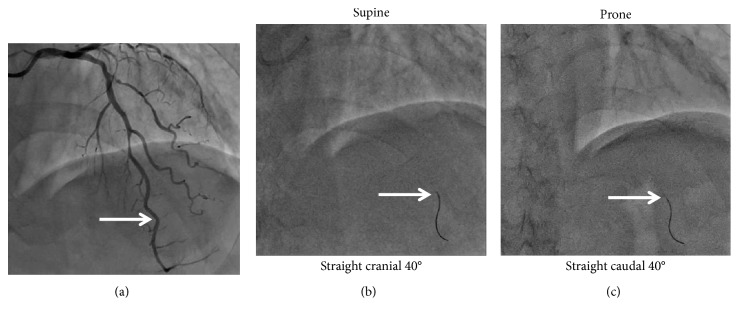
Intracoronary pressure measurements. FFR was measured in the supine position. Subsequently, patients were turned to a prone position. To ensure that the pressure wire's sensor is located in the same sites, side branches or tortuous parts were used as landmarks; then, FFR was similarly measured. FFR, fractional flow reserve.

Approach sites were the right or left radial arteries. Catheter examinations were performed using 4-French catheters (Goodtec JR4, JR (right radial), JL3.5, JL4, Goodman, Japan) after 3000-U heparin administration. The fourth intercostal axillary midline, which is the height of the right atrium, was taken as the zero point of the blood pressure line. The first cardiac catheterization was performed in the supine position. The pressure transducer was placed on the catheterization table level. A 0.014-inch pressure sensor-tipped wire (PressureWire™ X, St. Jude Medical, USA) was positioned at the guiding-catheter tip, and after intracoronary flushing with saline, pressure equalization was performed. The pressure wire was advanced into the target vessel for pressure recordings. The image of the position of the pressure wire was taken in the supine position. Resting mean aortic pressure (Pa) and mean distal intracoronary pressure (Pd) were recorded, and during hyperemia by intravenous administration of 180 *μ*g/kg/min adenosine, FFR was calculated as the ratio of Pd to Pa. The pressure wire sensor was retracted to the zero level to preclude pressure drift of the wire. In case of pressure drift of 0.01 or 0.02, Pd was corrected. In case of pressure drift >0.02, the measurement was discarded and another measurement was performed. Then, the pressure wire and catheter were removed. Subsequently, the patient was turned to a prone position. The pressure wire was similarly positioned at the guiding-catheter tip, and after intracoronary flushing with saline, pressure equalization was performed. The pressure wire sensor was advanced into the target vessel. To ensure that the pressure wire sensor sites were the same, while referring to the image in the supine position, we used the side branches or the tortuous parts as landmarks (Figure 1). The image of the position of the pressure wire in the prone position was also taken. Then, resting Pd/Pa and FFR were measured similarly. In cases of multiple lesions, resting Pd/Pa and FFR were measured in both positions in each vessel.

### 2.4. Height Differences between Pa and Pd Measurements by Heart CT

**Figure 2:**
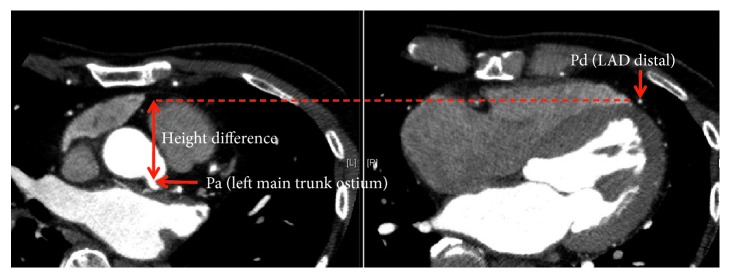
Height differences between Pa and Pd measurement by heart CT. Pa site was positioned at the ostium of the coronary artery on the CT image, and using side branches or tortuous parts as a landmark, Pd site was decided on the CT image. Two CT images matching the heights from the CT table were aligned, and the height differences between Pa and Pd were measured. CT, coronary tomography; Pa, mean aortic pressure; Pd, mean distal intracoronary pressure.

Metoprolol 20 mg was administered orally 2 hours before CT scan, and landiolol 0.125 mg/kg was additionally administered intravenously if necessary, targeting a heart rate <70 beats/min. All patients took nitroglycerin spray 0.3 mg just before the CT scan. Coronary CT was performed using a CT scanner with 80 detector rows (Aquilion PRIME SP, CANON Medical Systems Corporation, Tochigi, Japan). All scans were taken in the supine position with the patients holding their breaths at full inspiration. The following acquisition parameters were used: slice thickness 0.5 mm, tube voltage 120 kV, variable tube current 300–600 mA, rotation time 0.35 seconds, and pitch 0.175. All images were electronically retrieved on a workstation (SYNAPSE VINCENT FN-7941 Version 4.6.0003, FUJIFILM, Tokyo, Japan) and analyzed using an application (Coronary Analysis Version 4.6, FUJIFILM, Tokyo, Japan). The Pa site was positioned at the ostium of each coronary artery on the CT image, and using side branches or tortuous parts as a landmark, the Pd site was decided on the CT image. Two CT images matching the heights from the CT table were aligned, and height differences between Pa and Pd were measured (Figure 2).

### 2.5. Adjustment for Hydrostatic Pressure

A theoretical correction for resting Pd/Pa and FFR values was performed by adding physically expectable hydrostatic pressure of 0.077 mmHg per mm height difference to the distal coronary pressure wire sensor site (Pd), calculating the ratio of specific gravity of mercury (13.55 g/cm^3^) and blood (1.05 g/cm^3^) [14]. Height difference between the distal pressure sensor and catheter tip was used for adjustment in both positions.

### 2.6. Statistical Analyses

Continuous variables were presented as means with standard deviation and categorical variables as numbers and percentages. Resting Pd/Pa and FFR values were compared between the two positions using a paired *t*-test. Corrected FFR and resting Pd/Pa values in both positions were presented in scatter plots. Differences in FFR and resting Pd/Pa values between both positions and differences in corrected FFR and resting Pd/Pa values between both positions in LAD, LCX, and RCA were compared using a paired *t*-test.

Results were considered statistically significant at a *P* value < 0.05. All analyses were performed using SPSS PASW Statistics, Version 18.0 (Chicago, USA).

## 3. Results

### 3.1. Patient and Lesion Characteristics

Overall, 23 patients with 27 lesions were prospectively enrolled during the study period. Patient characteristics are summarized in Table 1. Mean age was 64.8 ± 9.3 years, and 83% of all patients were male. Lesion characteristics are summarized in Table 2. Only 2/27 vessels in this study were infarct vessels. Eleven lesions of LAD, 10 of LCX, and 6 of RCA were measured.

### 3.2. Height and Pressure Differences between Distal Pressure Sensor and Catheter Tip

**Figure 3:**
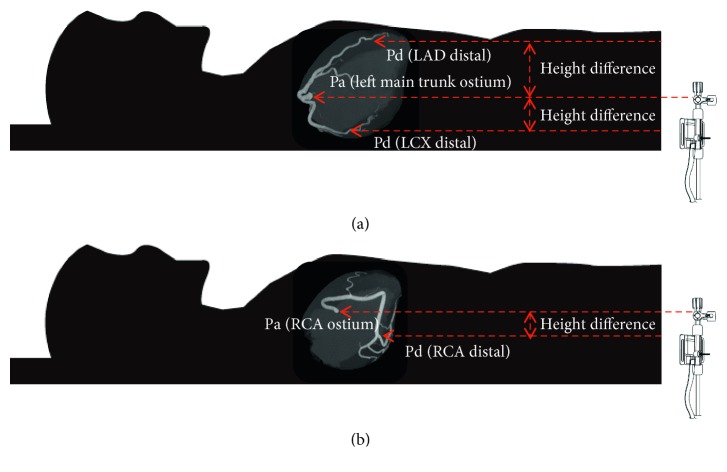
Anatomical position of LAD, LCX, and RCA. LAD takes an upward course, whereas LCX takes a downward course. RCA initially takes an upward course, runs horizontally, and then takes a downward course. LAD distal (Pd) is higher than the LMT ostium (Pa). LCX distal (Pd) is lower than the LMT ostium (Pa). RCA distal (Pd) is lower than the RCA ostium (Pa). LAD, left anterior descending artery; LCX, left circumflex artery; RCA, right coronary artery; LMT, left main trunk.

LAD takes an upward course, whereas LCX takes a downward course in the supine position. RCA initially takes an upward course, runs horizontally, and then takes a downward course.  Figure 3 shows the anatomical position of LAD, LCX, and RCA. In all 11 lesions of LAD, distal pressure sensors were located in the anterior position compared with the catheter tip location. Mean height difference (Pa–Pd) in LAD was −47.8 ± 14.6 mm, and mean pressure difference was 3.7 ± 1.1 mmHg. In all 10 lesions of LCX and all 6 lesions of RCA, the distal pressure sensor location was relatively posterior compared with the catheter tip location. Mean height difference (Pa–Pd) in LCX and RCA was +23.5 ± 8.5 mm and +29.2 ± 9.4 mm, respectively. Mean pressure difference in LCX and RCA calculated from the height difference was 1.8 ± 0.7 mmHg and 2.3 ± 0.7 mmHg, respectively. The mean bias of FFR, caused by hydrostatic pressure, was −0.046, +0.026, and +0.030 in LAD, LCX, and RCA, respectively. Detailed measurement data are presented in Table 3.

### 3.3. Resting Pd/Pa and FFR Values in the Supine and Prone Positions

**Figure 4:**
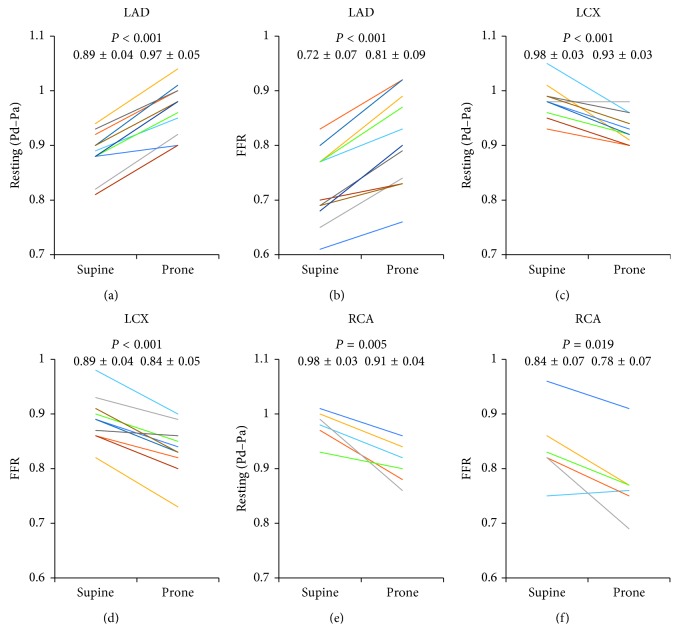
Resting Pd/Pa and FFR values in the supine and prone positions. Resting Pd/Pa and FFR values in the supine and prone positions in LAD (a, b), LCX (c, d), and RCA (e, f). Data are expressed as mean ± SD. LAD, left anterior descending artery; LCX, left circumflex artery; RCA, right coronary artery; SD, standard deviation; FFR, fractional flow reserve.

In LAD, resting Pd/Pa was significantly higher in the prone position than in the supine position (0.97 ± 0.05 vs 0.89 ± 0.04; *P* < 0.001) (Figure 4(a)) and the mean change was 0.08. FFR values were significantly higher in the prone position (0.81 ± 0.09 vs 0.72 ± 0.07; *P* < 0.001) (Figure 4(b)), and the mean change was 0.09. Conversely, in LCX, resting Pd/Pa and FFR values were significantly lower in the prone position (0.93 ± 0.03 vs 0.98 ± 0.03, *P* < 0.001 (resting Pd/Pa); 0.84 ± 0.05 vs 0.89 ± 0.04, *P* < 0.001 (FFR)) (Figures 4(c) and 4(d)), and the mean change for both values was 0.05. In RCA, resting Pd/Pa was significantly lower in the prone position (0.91 ± 0.04 vs 0.98 ± 0.03; *P*=0.005) (Figure 4(e)), and the mean change was 0.07. FFR values were significantly lower in the prone position (0.78 ± 0.07 vs 0.84 ± 0.07; *P*=0.019) (Figure 4(f)), and the mean change was 0.06.

### 3.4. Resting Pd/Pa and FFR Values Corrected by Hydrostatic Pressure in the Supine and Prone Positions

**Figure 5:**
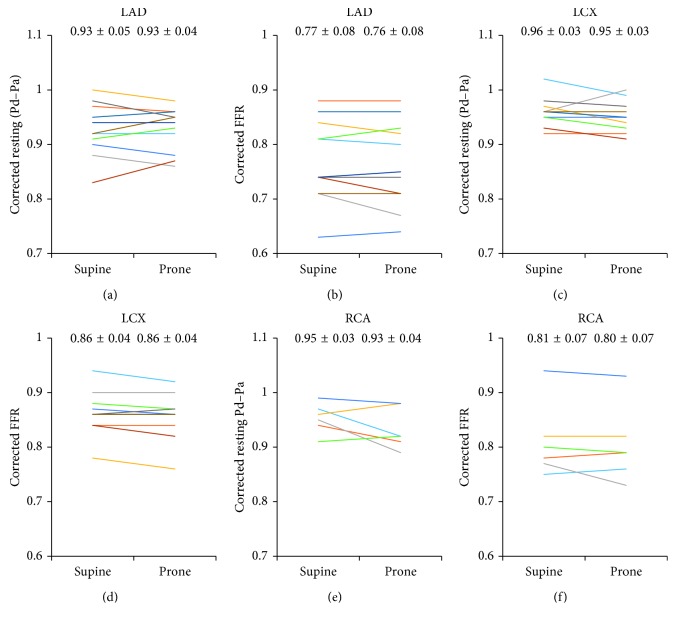
Resting Pd/Pa and FFR values corrected by hydrostatic pressure in the supine and prone positions. Resting Pd/Pa and FFR values corrected by hydrostatic pressure in the supine and prone positions in LAD (a, b), LCX (c, d), and RCA (e, f). Data are expressed as mean ± SD. LAD, left anterior descending artery; LCX, left circumflex artery; RCA, right coronary artery; SD, standard deviation; FFR, fractional flow reserve.

**Figure 6:**
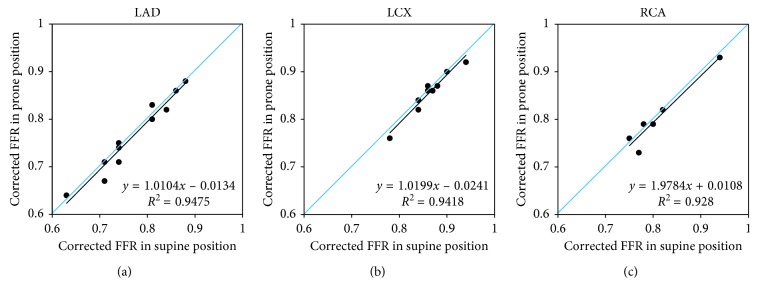
Comparison of FFR values corrected by hydrostatic pressure in the supine and prone positions. These plots show the linear regression analysis of FFR values corrected by hydrostatic pressure in the supine and prone positions in LAD (a), LCX (b), and RCA (c). LAD, left anterior descending artery; LCX, left circumflex artery; RCA, right coronary artery; FFR, fractional flow reserve.

Resting Pd/Pa and FFR values corrected by hydrostatic pressure in both positions were nearly equal (0.93 ± 0.04 vs 0.93 ± 0.05 and 0.76 ± 0.08 vs 0.77 ± 0.08, in LAD (Figures 5(a) and 5(b)); 0.95 ± 0.03 vs 0.96 ± 0.03 and 0.86 ± 0.04 vs 0.86 ± 0.04, in LCX (Figures 5(c) and 5(d)); 0.93 ± 0.04 vs 0.95 ± 0.03 and 0.80 ± 0.07 vs 0.81 ± 0.07, in RCA (Figures 5(e) and 5(f))). Good correlations were found between FFR values corrected by hydrostatic pressure in both positions (*R*^2^ = 0.948, in LAD (Figure 6(a)); *R*^2^ = 0.942, in LCX (Figure 6(b)); *R*^2^ = 0.928, in RCA (Figure 6(c))).

### 3.5. Differences in FFR Values between the Supine and Prone Positions and Differences in Corrected FFR Values between the Supine and Prone Positions

Compared with differences in FFR values between the supine and prone positions, those of the corrected FFR values were significantly lower in LAD, LCX, and RCA (0.08 ± 0.03 vs 0.01 ± 0.01, *P* < 0.001 for LAD; 0.06 ± 0.02 vs 0.01 ± 0.01 *P* < 0.001 for LCX; 0.07 ± 0.04 vs 0.01 ± 0.01 *P*=0.011 for RCA).

## 4. Discussion

The present study demonstrated that hydrostatic pressure variations due to height levels in Pa and Pd influence intracoronary pressure measurements and affect resting Pd/Pa and FFR values, by using measurements in the supine and prone positions in vivo. Our results revealed significantly lower values of resting Pd/Pa and FFR in the LAD supplying anterior coronary territories in the supine positions, whereas LCX or RCA with posterior coronary territories had significantly higher resting Pd/Pa and FFR values. Resting Pd/Pa and FFR values corrected by hydrostatic pressure in both positions were almost equal in LAD, LCX, and RCA.

The influence of hydrostatic pressure on the results of intracoronary pressure measurements was recently demonstrated in vitro [13]. The present study showed the influence of hydrostatic pressure using measurements in the supine and prone positions in vivo. The height difference between the catheter tip and distal pressure sensors was artificially changed by changing the patients' position on the table to investigate the hypothesized impact of hydrostatic pressure. In LAD, FFR values were significantly lower in the supine position than in the prone position, with differences as large as 0.09 on average. In LCX and RCA, FFR values were significantly higher in the supine position, with differences as large as 0.05 in LCX and 0.06 in RCA on average. These differences were too large to be ignored. To date, height difference and influence of hydrostatic pressure had been widely neglected. According to Pascal's law, hydrostatic pressure [*p*(*h*)] is the product of mass density (*ρ*), gravity (*g*=9.81 m/s^2^), and height difference (*h*) [*p*(*h*)=(*ρ* × *g* × *h*)]. Mass density of blood depends on plasma protein concentration, hematocrit [15], and temperature [16]. Under normal in vivo conditions, mass density of blood is approximately 1050 kg/m^3^ [17], leading to the expected hydrostatic pressure of 0.77 mmHg per cm height difference, similar to the results of the in vitro report [13]. On the basis of this physical law, FFR values were corrected by height differences in the supine and prone positions. Adjustment of hydrostatic pressure in the supine position increased all FFR values in LAD and decreased all values in LCX and RCA, whereas adjustment of hydrostatic pressure in the prone position decreased all FFR values in LAD and increased all values in LCX and RCA. Corrected FFR values were nearly equivalent in LAD, LCX, and RCA.

During extraction of the pressure wires in the healthy LAD in the supine positions, FFR values were often showed to gradually increase. In FFR measurement of LCX or RCA, resting Pd/Pa may reach >1.00 in some cases. These two phenomena may be explained by hydrostatic pressure. Given that the mean height differences were larger in LAD than in LCX or RCA, it was speculated that the influence of hydrostatic pressure between the supine and prone positions was larger in LAD than in LCX or RCA. In fact, the mean bias of FFR, caused by hydrostatic pressure, was −0.046, +0.026, and +0.030 in LAD, LCX, and RCA, respectively, in this study. The mean pressure differences calculated from height differences in LAD were 3.7 mmHg in our populations. When Pa is 100 mmHg, Pd is increased by 3.7 mmHg for adjustment and corrected FFR is increased by 0.037. If Pa is 50 mmHg, corrected FFR is increased by 0.074, which is twice the value of 0.037. The lower the blood pressure, the greater the influence on FFR value.

It was reported that from the analysis of coronary artery anatomy with 70 CTs, LAD takes an upward course, whereas LCX takes a downward course in all patients, RCA initially takes an upward course and then takes a downward course to the posterolateral branch (RPL), and the right posterior descending artery (RPD) takes an upward course again in the direction of the LV apex [13]. Since the distal pressure sensor is >30 mm away from the tip of the wire, during the measurement of RCA, the distal pressure sensor is around the RPL and RPD bifurcation, which is lower than the RCA ostium. If the pressure sensor site is at the proximal or middle RCA, the measurement is likely to have the same results in the two positions. This study might explain the systematic differences in FFR values, wherein LAD supplying the anterior coronary territories had lower FFR values, whereas LCX and RCA with posterior coronary territories had higher FFR values. This may have led to the report that only 24.2% of LAD lesions achieved optimal poststent FFR (FFR ≥ 0.9), compared with 69.0% of LCX lesions and 70.4% of RCA lesions [18], and the results of the reverse mismatch in LAD and mismatch in LCX and RCA [10–12]. There is a possibility that the culprit may be mistaken as the moderate stenosis in LAD instead of the severe stenosis in the LCX or RCA.

Although several reports have demonstrated that noninvasive FFR derived from CT and invasive FFR had a good correlation, these have not been compared in LAD, LCX, and RCA [19, 20]. Recently, methods not requiring patients to be in the state of hyperemia, such as instantaneous wave-free ratio or diastolic pressure ratio, have been developed. The diastolic pressure ratio at rest, calculated using novel software applicable to any type of pressure wire, was introduced as alternative invasive indices to assess coronary artery stenosis severity [21, 22]. Inducing a hyperemic state is not required; thus, the frequency of its use may increase in the future. However, diastolic coronary artery pressure is usually lower than the average coronary artery pressure; thus, hydrostatic pressure may be more influential. Interventional cardiologists must be aware that measurement results in LAD do not represent the same functional effect of a stenosis when compared with the results in posterior vessels. Therefore, other methods of diagnosing ischemia are required for comprehensive evaluations.

The cutoff FFR values such as those reported in FAME [2, 4, 6, 7] and DEFER study [5, 23] are all combined without dividing LAD, LCX, and RCA. The cutoff FFR values may need to be examined separately by LAD, LCX, and RCA or be examined using FFR values corrected by hydrostatic pressure, which could be calculated by 0.77 mmHg per cm height difference. The height difference between the catheter tip and distal pressure sensor can be measured in the left lateral view using autocalibration of the X-ray system. It may be necessary to confirm the effect of this hydrostatic pressure and to re-evaluate the cutoff value in a prospective clinical trial in a large population. Using corrected FFR values by hydrostatic pressure may result in a stronger correlation with other methods for evaluating myocardial ischemia, such as SPECT perfusion imaging or FFR derived from coronary CT angiography than uncorrected FFR values.

According to the pivotal study, FFR is the relation of the difference between coronary pressure distal to a stenosis (Pd) and mean central venous pressure (Pv) and the difference between mean aortic pressure (Pa) and Pv [FFR = (Pd − Pv)/(Pa − Pv)] [24]. For reasons of simplification, venous pressure was neglected in the FFR calculation in the course [1, 25]. It is known that changing from the supine position to the prone position results in significant differences in central venous pressure [26, 27].

This study has several limitations. First, the number of cases was small; thus, the measured differences did not deny the abundant data reported so far in many studies on FFR. Second, the height differences were measured using cardiac CT, which is not perfectly accurate. Third, when turning the patient to the prone position, the height difference may not be opposite to that in the supine position. Fourth, it is unclear whether a particular group of patients (those with obesity and emphysema) can influence the differences shown between the supine and prone positions. Fifth, as the catheter and pressure wire were reinserted, the distal sensor site may be different between both positions. Given that we had to pay attention to both positions of the pressure wire and catheter tip, which can be removed easily while inserting and conversely entering deep into the coronary artery during withdrawal, it may be difficult to achieve precisely similar measurement positions. Sixth, in our study, we used a standard value of 1.05 as the mass density of blood. Seventh, as FFR was measured using a 4-Fr catheter, measurements might be inaccurate compared with that when using 5-Fr or larger catheters.

## 5. Conclusion

Our results revealed significantly lower values of resting Pd/Pa and FFR in LAD supplying the anterior coronary territories in the supine positions, whereas LCX or RCA with posterior coronary territories had significantly higher resting Pd/Pa and FFR values. Cutoff FFR values may need to be separately examined by LAD, LCX, and RCA or be examined using FFR values corrected by hydrostatic pressure in future studies.

## Figures and Tables

**Table 1:** Patient characteristics.

	*N = *23
Age (years)	64.8 ± 9.3
Male	19 (83%)
Hypertension	14 (61%)
Diabetes mellitus	8 (35%)
Dyslipidemia	12 (52%)
Smoking	5 (22%)
Family history	2 (9%)
Chronic kidney disease	4 (17%)
Hemodialysis	0 (0%)
Congestive heart failure	1 (4%)
Old myocardial infarction	10 (43%)
Previous PCI	8 (35%)
Previous CABG	2 (9%)
EF (%)	62.1 ± 12.3
Peripheral artery disease	0 (0%)
Old cerebral infarction	1 (4%)
COPD	0 (0%)

Number of disease vessels	
0	1 (4%)
1	10 (43%)
2	12 (52%)
3	0 (0%)

Medication	
Antiplatelet agent	14 (61%)
Anticoagulation	2 (9%)
Beta-blocker	6 (26%)
Renin-angiotensin system inhibitors	10 (43%)
Statin	14 (61%)
Calcium channel blocker	6 (26%)
Oral diabetes drugs	3 (13%)
Insulin	1 (4%)

Data are expressed as mean ± SD and numbers (%). COPD, chronic obstructive pulmonary disease; EF, ejection fraction; PCI, percutaneous coronary intervention; CABG, coronary artery bypass graft.

**Table 2:** Lesion characteristics.

	*N = *27
Diagnosis	
Stable angina	14 (52%)
Silent myocardial ischemia	11 (41%)
Old myocardial infarction	2 (7%)

Lesion	
LAD	11 (41%)
LCX	10 (37%)
RCA	6 (22%)

Type	
A/B1	21 (78%)
B2/C	6 (22%)

Data are expressed as numbers (%). LAD, left anterior descending artery; LCX, left circumflex artery: RCA, right coronary artery.

**Table 3:** Height and pressure differences between distal pressure sensor and catheter tip.

	Height differences (Pa–Pd) (mm)	(mmHg)
LAD (*n*=11)	−47.8 ± 14.6	3.7 ± 1.1
LCX (*n*=10)	+23.5 ± 8.5	1.8 ± 0.7
RCA (*n*=6)	+29.2 ± 9.4	2.3 ± 0.7

Data are expressed as mean ± SD. In the position where Pd is higher than Pa, it is represented by a minus sign, whereas in the position where Pd is lower than Pa, it is represented by a plus sign. LAD, left anterior descending artery; LCX, left circumflex artery; RCA, right coronary artery; Pa, mean aortic; Pd, mean distal intracoronary pressure.

## Data Availability

The data used to support the findings of this study are included within the supplementary information file.

## Supplementary Materials

Supplementary Materials The supplementary material file contains the data of patient characteristics, lesion characteristics, and height differences between distal pressure sensor and catheter tip on CT. COPD, chronic obstructive pulmonary disease; EF, ejection fraction; PCI, percutaneous coronary intervention; CABG, coronary artery bypass graft LAD, left anterior descending artery; LCX, left circumflex artery: RCA, right coronary artery; AP, angina pectoris; OMI, old myocardial infarction; SMI, silent myocardial ischemia.
